# Suspected ventilator-associated respiratory infection in severely ill patients: a prospective observational study

**DOI:** 10.1186/cc13077

**Published:** 2013-10-22

**Authors:** Jason Shahin, Michael Bielinski, Celine Guichon, Catherine Flemming, Arnold S Kristof

**Affiliations:** 1 Department of Critical Care, McGill University Health Centre, Montreal, Canada; 2 Department of Medicine, Respiratory Division, McGill University Health Centre, Montreal, Canada; 3 McGill University Health Centre, Royal Victoria Hospital Site, 687 Pine Ave. W., L3.05, Montreal QC H3A 1A1, Canada

## Abstract

**Introduction:**

Ventilator-associated respiratory infection (VARI) is an important cause of morbidity in critically-ill patients. Clinical trials performed in heterogeneous populations have suggested there are limited benefits from invasive diagnostic testing to identify patients at risk or to target antimicrobial therapy. However, multiple patient subgroups (for example, immunocompromised, antibiotic-treated) have traditionally been excluded from randomization. We hypothesized that a prospective surveillance study would better identify patients with suspected VARI (sVARI) at high risk for adverse clinical outcomes, and who might be specifically targeted in future trials.

**Methods:**

We performed a prospective observational study in all patients ventilated for greater than 48 hours. sVARI was identified by surveillance for changes in white blood cell count, temperature, sputum, and/or new chest X-ray infiltrates. Indices of disease co-morbidity, as well as mortality, duration of mechanical ventilation, and length of hospital or ICU stay were correlated with sVARI.

**Results:**

Of 1806 patients admitted to the ICU over 14 months, 267 were ventilated for greater than 48 hours, and 77 developed sVARI. Incidence of sVARI was associated with iatrogenic immunosuppression or admission for respiratory illness. Any sVARI, whether suspected ventilator-associated pneumonia (sVAP) or ventilator-associated tracheobronchitis (sVAT), was associated with increased length of stay and duration of mechanical ventilation.

**Conclusions:**

Clinical surveillance for sVARI identifies patients at risk for increased morbidity. Iatrogenically immunosuppressed patients, a subgroup previously excluded from randomized clinical trials, represent a growing proportion of the critically-ill at risk for sVARI who might be targeted for future investigations on diagnostic or therapeutic modalities.

## Introduction

Ventilator-associated respiratory infection (VARI), which includes ventilator-associated pneumonia (VAP) and ventilator-associated tracheobronchitis (VAT), is a common acquired infection in the ICU that contributes to excess length of stay, duration of ventilation, cost and likely mortality [1,2]. The pathogenesis is thought to involve predisposition to microbial colonization and aspiration of pathogenic organisms from the upper respiratory tract [2]. In agreement, prevention strategies that minimize colonization and aspiration (for example, anti-microbial-coated endotracheal tubes, semi-erect feeding) have significantly reduced the incidence of VAP [1]. However, the associated morbidity, antibiotic usage and cost remain high [3]. Many have advocated the use of invasive lower respiratory tract sampling to target pathogenic organisms better and to limit the inappropriate use of antibiotics. Nonetheless, in part due to the poor predictive value of microbial cultures, current guidelines reflect ambivalence regarding the utility of lower respiratory tract sampling and/or quantitative cultures in the management of VAP [1,4].

The use of invasive strategies to improve diagnostic accuracy and prescribe targeted therapies was examined in two large trials. In one study, quantitative cultures of lower respiratory tract samples resulted in reduced mortality, antibiotic use and organ failure [5]. In a more recent trial, there was no survival benefit for the use of invasive techniques [6]. A systematic review based on these, and two other small clinical trials [7-10], suggested that there is no effect of invasive sampling or quantitative cultures on mortality in patients with suspected VAP. However, the trials excluded the majority of screened patients, or those patients with risk factors commonly encountered in contemporary quaternary care ICUs (for example, iatrogenic immunosuppression, previous bacterial colonization, concurrent use of broad spectrum antibiotics).

Since exclusion of patients at high risk may lead to underestimation of the effects of interventions on clinical outcomes, we questioned whether the study populations reflect those patients encountered in current quaternary care clinical practice. We hypothesized that a prospective analysis of patients at risk for VARI might better identify associated risk factors independent of respiratory tract samples for bacterial diagnosis, and that VAP need not be distinguished from VAT. Here, we identify patient subgroups at higher risk of morbidity that can be targeted, and not excluded, in future clinical trials on diagnostic and therapeutic modalities in the personalized treatment of VARI independent of bacteriological diagnosis.

## Materials and methods

### Study population

Over a period of 14 months (1 May 2007 to 31 July 2008), all patients in the ICU unit at the McGill University Hospital Centre who were mechanically ventilated for longer than 48 hours were prospectively identified on a daily basis. All study patients were more than 18 years old. The study was reviewed and approved by the McGill University Health Centre research ethics board. Data were collected by prospective or retrospective chart review or database search, with permission from the hospital Director of Professional Services, and as dictated by the approved ethics protocol. Patient consent was not required for the use of anonymous chart-based clinical or laboratory data.

### Data collection

Prospective data collection for the identification of suspected VARI (sVARI) included the highest temperature in the last 24 hours, white blood cell count (WBC) and neutrophil count, as well as increased quantity of respiratory secretions. Sputum was characterized as being of small, moderate, or large amounts. For patients with suspected VARI, chest X-ray or computed tomography (CT) chest changes compatible with pneumonia at the time of surveillance were identified by two physicians and a radiologist by inspection of digital images or reports. In addition, at the time of surveillance, we recorded the duration of mechanical ventilation in days and antibiotic or immunosuppressive medications. After prospective data collection had terminated, we extracted the following parameters for each patient from our hospital clinical database: age, sex, primary diagnosis at admission, associated co-morbidities (see Table 1), Acute Physiology and Chronic Health Evaluation II (Apache II) score [11], mortality, total duration of mechanical ventilation, length of stay in ICU, and length of stay in hospital. When available, we retrieved results of upper respiratory tract bacterial cultures for patients with sVARI.

**Table 1:** Baseline clinical characteristics of the total cohort and those with suspected ventilator associated respiratory infection (sVARI)

	**Total cohort (267)**	**No VARI (190)**	**Suspected VARI (77)**	***P *** **value**
**Median age (IQR)**	55 (46 to 65)	55(46 to 67)	55(46 to 66)	0.77
**Male sex (%)**	166 (62.7)	116(61.1)	50(64.9)	0.55
**Primary diagnosis at admission**^**a**^				
** Respiratory**	51 (18.8)	30(15.8)	20(26.0)	0.05
** Cardiovascular**	8 (3.0)	6(3.2)	2(2.6)	0.81
** Gastroenterological**	20 (7.4)	14(7.4)	6(7.8)	0.91
** Neurological**	3 (1.11)	3(1.6)	0	0.27
** Sepsis**	45 (16.6)	29(15.3)	16(20.8)	0.28
** Post-operative**	109 (40.2)	79(41.6)	28(36.4)	0.43
** Post arrest**	8 (3.0)	8(4.2)	0	0.07
** Other**	8 (3.0)	14 (7.4)	5(6.5)	0.80
**Co-morbidities**				
** Diabetes**	92 (34.0)	64(33.7)	26(33.8)	0.99
** COPD/Asthma**	51 (18.8)	34(17.9)	17(22.1)	0.43
** CAD**	132 (48.7)	94(49.5)	36(46.8)	0.69
** CHF**	77 (28.4)	52(27.4)	25(32.5)	0.41
** Liver disease**	54 (19.9)	38(20.0)	15(19.5)	0.92
** Malignancy**	73 (26.9)	47(24.7)	25(32.5)	0.20
** Dementia**	9 (3.3)	5(2.6)	4(5.2)	0.3
** Immunosuppressed**	34 (12.7)	17 (9.0)	17(22.1)	0.004
** Stroke**	29 (10.7)	23(12.1)	6(7.8)	0.31
**CRF**	75 (27.7)	52(27.4)	23(29.9)	0.68
**APACHE II Score**^**b**^** (IQR)**	27 (22 to 34)	27(21 to 36)	27(22 to 33)	0.83

^a^Nineteen patients had missing initial diagnoses; ^b^APACHE scores available for 64% of the cohort. The remainder of patients consisted of cardiac surgery admissions for which the APACHE score has not been validated. APACHE II, Acute Physiology and Chronic Health Evaluation II; COPD, chronic obstructive pulmonary disease; CAD, coronary artery disease; CHF, congestive heart failure; CRF, chronic renal failure; IQR, interquartile range.

### Definitions

For each patient, sVARI was defined as the first occurrence of suspected VAP (sVAP) or suspected VAT (sVAT). sVAP was defined as a change in the chest X-ray consistent with pneumonia, and two of the following: WBC ≥11 or <4, temperature ≥38.0 or <36.0°C, or an increase in sputum. sVAT was defined as an increase in sputum with WBC ≥11 or <4, and temperature ≥38.0 or <36.0°C (adapted from [12]). Iatrogenically immunosuppressed patients were identified prospectively as those who were administered immunosuppressive medications (for example, calcineurin inhibitors, anti-metabolite DNA synthesis inhibitors, lymphotoxic agents, mTOR inhibitors, high-dose steroids) on the day of surveillance for sVARI. Other conditions associated with relative immunosuppression (for example, diabetes, cancer, liver disease, chronic renal failure) were accounted for in multivariable analyses. We did not adjust for rare cases of chemotherapy-induced neutropenia, as neutropenia was a criterion for the diagnosis of sVARI.

### Statistical analysis

We analyzed all baseline characteristics for the total cohort and for groups of patients without or with sVARI. We explored all variables with frequency distributions and cross tabulations. For normally distributed continuous data, results were expressed as means ± standard deviation and compared using two-tailed Student’s *t*-test. For skewed continuous data, results were expressed as medians ± interquartile range (IQR) and compared using the Wilcoxon rank sum test. Categorical variables were compared using a Chi-squared test.

A multivariable analysis was conducted using logistic regression to assess the association between immunosuppression and sVARI. In order to adjust for confounding, *a priori* chosen covariates were included in the model. No statistical selection process was used for model covariate selection. Specifically, we adjusted for age, diabetes, liver disease, malignancy and chronic renal failure. APACHE II score was not included due to the inclusion of a large proportion of cardiac surgery patients, for which the APACHE II score was not originally validated. Multiple linear regression was used to assess the association between skewed continuous variables (for example, ICU length of stay, hospital length of stay) and sVARI while adjusting for *a priori* covariates. To account for the skewed nature of the data the continuous outcome variable was log transformed prior to model development. Statistical analyses were performed using Stata Version 10.1 (StataCorp LP, College Station TX, USA).

## Results

**Figure 1:**
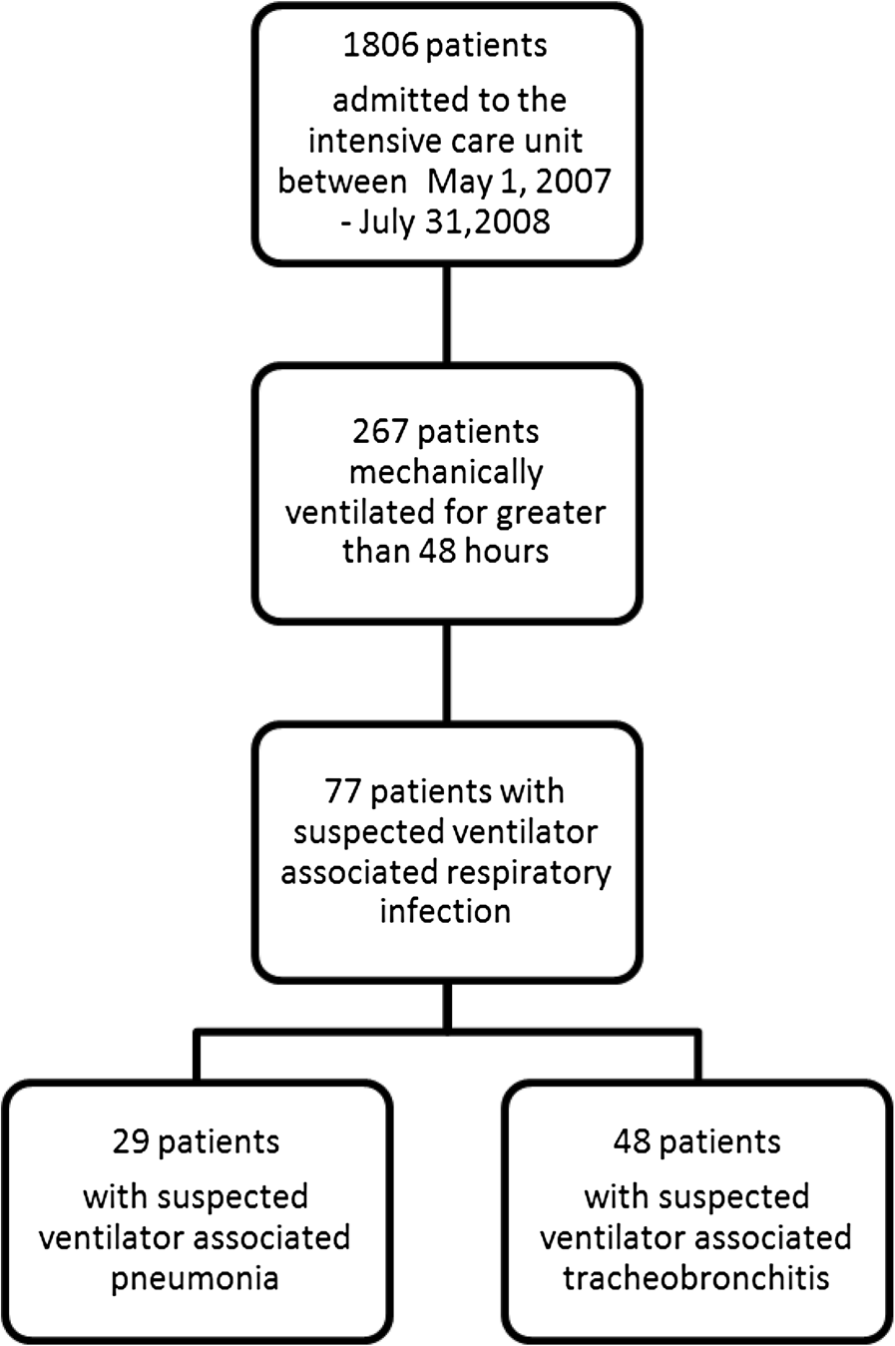
**Patient flow.** A flow diagram for patients at risk for suspected ventilator-associated pneumonia, and identification of patients with suspected ventilator-associated tracheobronchitis (sVAT) or suspected ventilator-associated pneumonia (sVAP), are shown.

Over the course of 14 months, 1,806 patients were admitted to the ICU, and in 287 (14.8%) of these the duration of mechanical ventilation was more than 48 hours. There was a total of 3,202 ventilator days or 2,068 ventilator days per 1,000 patients per year. The median duration of mechanical ventilation was 10 days (IQR, 5 to 15 days). During the study period, 77 patients developed sVARI (Figure 1). Of the 77 patients with sVARI, 62% had sVAT and 38% sVAP. For the 62 respiratory tract microbial cultures retrospectively retrieved in patients with sVARI, the spectrum of organisms was similar to that observed in other quaternary care ICUs (see Additional file 1). The median time to sVARI was five days (IQR, three to eight days), and one third occurred within three days of initiation of mechanical ventilation (see Additional file 2).

We determined baseline demographics, as well as indicators of diagnostic spectrum or severity of illness (Table 1). The median age was 55 years, and two thirds of the patients were men. The most common reason for admission was for post-operative monitoring after complicated surgery or intra-operative complications. Respiratory illness or sepsis were also common diagnoses, and together accounted for one third of admissions to the ICU. The most frequent comorbidities were diabetes and coronary artery disease. Active malignancy and iatrogenic immunosuppression (referred to as immunosuppression from here on) were also common and occurred in 26.9% and 12.7% of patients, respectively. The median APACHE II score was 27 (IQR, 22 to 34).

In immunosuppressed patients, sVARI occurred more commonly than no sVARI (22.1% *versus* 9%, *P* = 0.004). Other than patients admitted with respiratory disease (sVARI 26% *versus* no sVARI 15.8%, *P* = 0.05), there were no significant differences for other co-morbidities listed in Table 1. Of the 77 patients with a new diagnosis of sVARI, 51 (66%) were concurrently undergoing administration of broad spectrum antibiotics. Patients with sVARI had increased duration of mechanical ventilation, ICU and hospital length of stay, but not mortality, when compared to patients with no infection (Table 2). When compared to non-immunocompromised patients with sVARI, those who were immunocompromised exhibited increased median ICU length of stay (26 days, IQR 20 to 44 *versus* 16 days, IQR 11 to 31; *P* = 0.08) and hospital length of stay (113 days, IQR 76 to 154 *versus* 46 days, 95% CI 29 to 91; *P* <0.002), but not mortality (47% *versus* 35%; not significant) or duration of mechanical ventilation (9 days, IQR 4 to 15 *versus* 9 days, IQR 6 to 15; not significant). In a multivariable analysis, immunosuppression was significantly associated with sVARI even after adjusting for other chronic diseases associated with reduced immune function (odds ratio 3.34, 95% CI 1.47 to 7.61, *P* = 0.004) (Table 3).

**Table 2:** Clinical outcomes of total cohort and patients with and without sVARI

	**Total cohort (271)**	**No VARI (238)**	**Suspected VARI (77)**	***P***^**a **^**Value**
**ICU mortality number (%)**	92 (34.1)	63(33.3)	29(37.7)	0.50
**Median number of days on ventilator (IQR)**	6 (4 to 12)	6(3 to 10)^b^	9(6 to 15)	0.0002
**Median number of days in ICU (IQR)**	12 (7 to 23)	11(7 to 20)	18(11 to 36)	<0.001
**Median number of days in hospital (IQR)**	39 (19 to 80)	34(16 to 69)	60(33 to 129)	<0.001

^a^*P* value relates to patients with and without suspected VARI; ^b^21 patients (11%) with missing data for vent days. IQR, interquartile range; VARI, ventilator-associated respiratory infection.

**Table 3:** Association between immunosuppression and sVARI by multivariable analysis

**Baseline characteristics**	**Odds ratio**	**Confidence interval (95%)**	***P*-value**
**Immunosuppressed**	3.34	1.47 to 7.61	0.004
**Age**			
** <50**	1.0	-	
** 50 to 70**	0.78	0.34 to 1.79	0.36
** >70**	0.89	0.38 to 2.11	
**Diabetes**	0.94	0.51 to 1.73	0.87
**Liver disease**	0.78	0.37 to 1.61	0.50
**Malignancy**	1.30	0.71 to 2.39	0.85
**CRF**	0.92	0.51 to 1.73	0.80

CRF, chronic renal failure; sVARI, suspected ventilator-associated respiratory infection.

We next determined the distribution of sVARI sub-types, and their effects on patient outcomes. Forty-eight (62%) patients with sVARI had tracheobronchitis (sVAT) (see Additional file 1). Of the 29 (38%) patients with sVAP, 19 exhibited abnormal white blood cell count or sputum production, but not temperature. Only three patients with sVARI developed all four diagnostic criteria for sVAP. These data indicate that the classical signs and symptoms associated with VAP are often absent, and that sVAT occurs in a large proportion (62%) of patients with sVARI.

**Figure 2:**
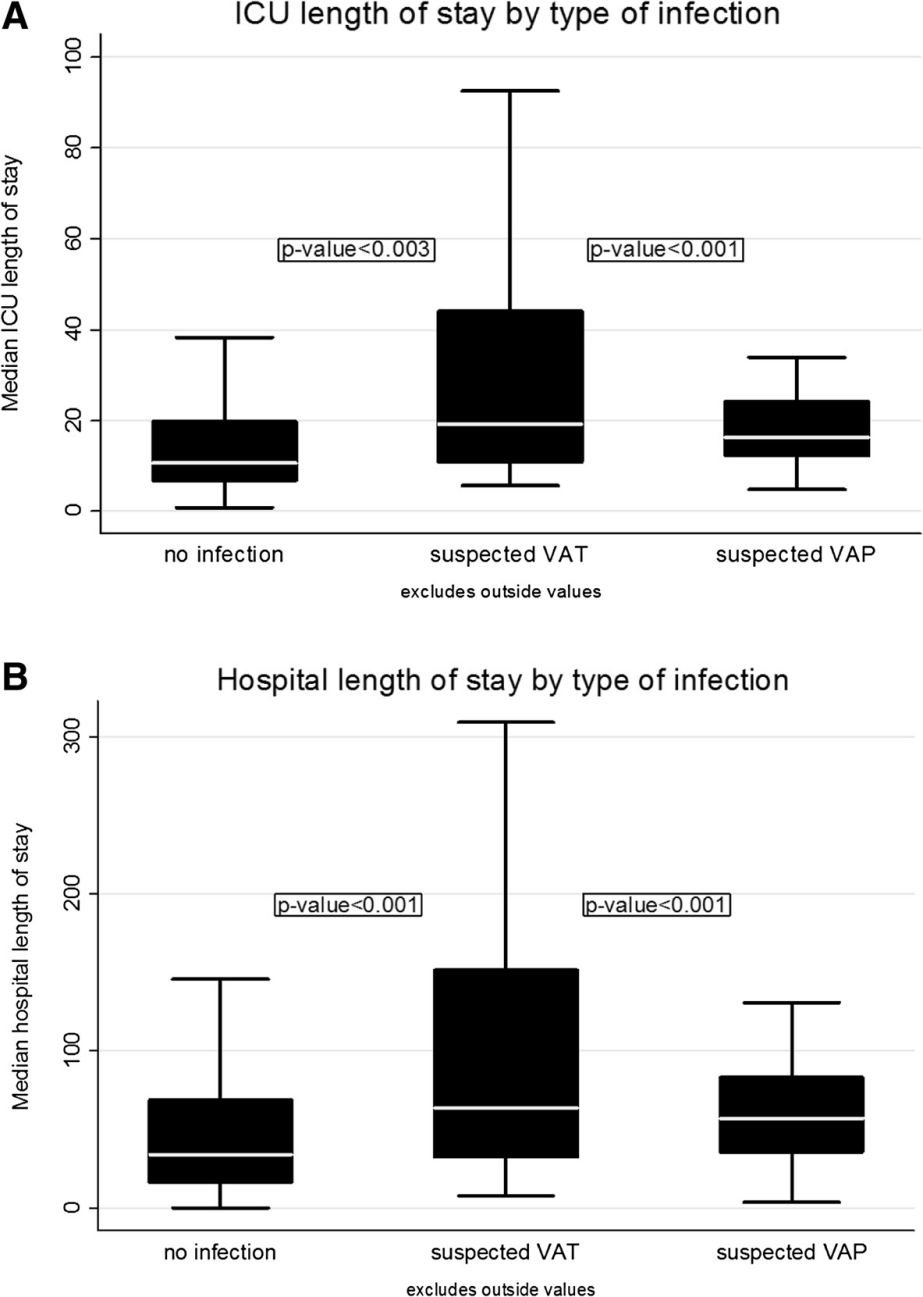
**ICU or hospital length of stay in patients with sVAP or sVAT.** For patients with no suspected infection, sVAT or sVAP, median values ± interquartile range (IQR) are shown for **A)** ICU length of stay (days) or **B)** hospital length of stay (days). *P* <0.003 for sVAT or sVAP *versus* no infection by Wilcoxon rank sum test. sVAP, suspected ventilator-associated pneumonia; sVAT, suspected ventilator-associated tracheobronchitis.

We therefore determined whether sVAP and sVAT differed in their effects on ICU or hospital length of stay. When compared with patients with no infection, those with sVAP had a significantly increased ICU (16 days, IQR 12 to 24 *versus* 11 days, IQR 7 to 20, *P* <0.01) or hospital (57 days, IQR 35 to 83 *versus* 34 days, IQR 16 to 69, *P* <0.01) length of stay (Figure 2). Patients with sVAT had increased ICU (19 days, IQR 11 to 44 *versus* 11 days, IQR 7 to 20, *P* <0.003) or hospital (64 days, IQR 32 to 152 *versus* 34 days, IQR 16 to 69, *P* <0.001) length of stay. In a separate multivariable analysis, after adjusting for immunosuppressive medications, age, diabetes, liver disease, cancer and chronic renal failure, there was a 1.8-, 2.0-, and 1.5-fold increase in ICU length of stay for patients with sVARI, sVAT, and sVAP, respectively (each *P* <0.001). Similarly, there was a 1.6-, 1.8-, and 1.6-fold increase in hospital length of stay for patients with sVARI, sVAT and sVAP, respectively (*P* <0.001). These data indicate a significant effect of sVAP and sVAT on length of stay even when adjusting for immunosuppression.

## Discussion

In this study, we assessed the incidence, risk factors and clinical outcomes of sVARI over a 14 month period in the 267 patients who were ventilated for more than 48 hours in a quaternary care ICU. The diagnosis of sVARI incorporated ventilator-associated complications independent of bacteriological diagnosis and was associated with iatrogenic immunosuppression (that is, patients on immunosuppressive agents at the time of surveillance for sVARI) and respiratory illness, as well as an increased duration of mechanical ventilation, or length of stay in the ICU or hospital. Whereas most studies have focused on VAP, we found that sVAT was also associated with increased ICU or hospital length of stay. The rate of sVAT (18%) was slightly higher than that previously reported for VAT (that is, that incorporating bacterial culture results) [13]; a recent meta-analysis reported a frequency of 11.5% [14]. In support of the clinical importance of VAT, VAT and VAP had similar impacts on outcomes in a study where bacterial cultures were taken into account [13]. Moreover, anti-bacterial treatment of VAT increased mechanical ventilation-free days and survival in a small randomized trial [15]. Although our results suggest that clinical indicators of ventilator-associated respiratory tract infection identify patients at risk for adverse outcomes, the effect of respiratory tract sampling on patient outcomes or anti-bacterial usage in high-risk patients (for example, immunocompromised), or those already undergoing antibiotic therapy, remains unknown.

We report that iatrogenically immunosuppressed patients are at increased risk for VARI, which in turn impacts upon ICU and hospital length of stay. In agreement, patients with sVARI who were immunocompromised exhibited an increased duration of ICU and hospital stay when compared to those who were not immunocompromised. Consistent with our observations, a large surveillance study revealed that immunosuppression is an independent predictor of all infections in the ICU and more than 50% of infections were respiratory [16]. This subgroup of patients has been traditionally excluded from randomized trials on VAP. Moreover, in recent years, the use of immunosuppressive agents for transplantation or chronic inflammatory disease has dramatically increased, and this group of patients now represents a significant proportion of quaternary ICU admissions. Unlike randomized trials with heterogeneous patient populations [6], targeting a limited population at increased risk might shed light on the specific effects of lower respiratory tract sampling and microbial ecology on clinical outcomes in future studies.

Our prospective trial design permitted the assessment of sVARI on clinical outcomes independent of biases introduced by retrospective chart review. The large number of patients with solid organ and bone marrow transplantation in our ICU permitted the evaluation of factors associated with immunosuppression, which may have been missed by previous surveillance studies [12]. In fact, patients with active malignancy or immunosuppression represented 40% of our cohort. Reassuringly, demographics (for example, age, co-morbidities, admission diagnosis; Table 1) and microbial ecology (see Additional file 1) were comparable to previous studies on ventilator-associated infections, with the exception of patients with trauma, burn and primary neurological presentations. Consistent with previous studies, a primary respiratory diagnosis on admission was associated with sVARI [2]. Median duration of mechanical ventilation, APACHE II scores, rates of ventilator-associated complications, mortality and length of ICU or hospital stay (Tables 1 and 2) were also similar to those in other reports on patients who were ventilated for >48 hours [17]. Moreover, as previously reported [13], the majority of ventilator-associated events occurred within six days of initiation of mechanical ventilation (see Additional file 2).

We detected a significant association between immunosuppression and sVARI (Table 3, odds ratio 3.3, IQR 1.5 to 7.6). In addition, patients with sVARI exhibited increased ICU and hospital length of stay if they were immunosuppressed. However, due to a relatively low event rate (77 patients with sVARI), one limitation of our study was suboptimal power to rule out differences between certain groups, or to use our dataset to discover new markers of sVARI or related adverse clinical outcomes. Moreover, although the spectrum of organisms was similar to that observed in other studies on VAP (see Additional file 1), we did not collect microbiological samples for all patients and time points; we could, therefore, not assess the relationship between type of bacterial infection and demographic characteristics or clinical outcomes. With regard to the latter, many would argue that poor standardization and specificity renders bacteriological diagnosis suboptimal for the diagnosis of VAP or VAT, although they may be useful for surveillance (http://www.cdc.gov/nhsn/acute-care-hospital/vae). This may, in part, be due to the common use of broad spectrum antibiotics at the time of surveillance. In the current study, 66% of the patients were on broad-spectrum antibiotics at the time of sVARI. Similar antibiotic usage rates have been demonstrated in previous studies [13,15,16]. Importantly, patients on prior antibiotics were excluded from a negative trial evaluating lower respiratory tract sampling in VAP [6].

## Conclusions

In summary, mechanically-ventilated patients at risk for adverse clinical outcomes can be identified on clinical grounds. Patients at risk include those who develop suspected VAP or VAT (that is, sVARI) and those who are iatrogenically immunosuppressed. Observational studies with larger sample sizes can be performed to rule out other risk factors for sVARI that were not identified in our study as statistically significant. Iatrogenically immunosuppressed patients represent an emerging population at risk for VARI that can be targeted in future randomized trials on the role of diagnostic or therapeutic modalities in ventilator-associated complications. Although there is a current emphasis on the identification of ‘ventilator-associated events (VAEs)’ as quality indicators in hospital-based health care delivery (http://www.cdc.gov/nhsn/acute-care-hospital/vae), our data highlight the importance of suspected VARIs as an important outcome for the development of targeted diagnostic and therapeutic strategies.

## Key messages

•Suspicion of ventilator-associated respiratory infection based on clinical criteria is associated with iatrogenic immunosuppression in critically ill patients.

•Suspicion of ventilator-associated pneumonia or tracheobronchitis is equally associated with adverse clinical outcomes.

•We identified patient groups at high risk for adverse outcomes that can be targeted in future clinical trials on diagnostic and therapeutic modalities for ventilator-associated infections.

•Despite the current enthusiasm for a focus on ventilator-associated events as an outcome for hospital-acquired infections, VARIs remain important for identifying patients who might derive benefit from targeted diagnostic or therapeutic modalities.

## Abbreviations

APACHE II: Acute Physiology and Chronic Health Evaluation II; CXR: Chest X-ray; IQR: Interquartile range; sVAP: suspected ventilator-associated pneumonia; sVARI: suspected ventilator-associated respiratory infection; sVAT: suspected ventilator-associated tracheobronchitis; VAP: Ventilator-associated pneumonia; VARI: Ventilator-associated respiratory infection; VAT: Ventilator-associated tracheobronchitis; WBC: White blood cells.

## Competing interests

The authors declare that they have no competing interests.

## Authors’ contributions

JS supervised and carried out the data analysis and participated in drafting the manuscript. MB participated in retrospective data collection, coordination, and analysis, as well as drafting of the manuscript. CG participated in data analysis as well as preparation of the manuscript. CF collected the prospective data and participated in database construction. AK conceived of and designed the study and coordinated drafting of the manuscript. All authors read and approved the final manuscript.

## Supplementary Material

Additional file 1: Table S1 Organisms isolated in patients with suspected ventilator-associated respiratory infection; **Table S2**: Breakdown of diagnostic criteria for patients with sVARI. Click here for file

Additional file 2 **Frequency distribution of sVARI by days of mechanical ventilation.** Data shown are a frequency distribution representing the number of incident sVARIs by duration of mechanical ventilation. Click here for file
