# Supplementary material for: Suspected ventilator-associated respiratory infection in severely ill patients: a prospective observational study
Source: Crit Care. 2013 Oct 22;17(5):R251. doi: 10.1186/cc13077 (PMC4056611; doi:10.1186/cc13077)
Supplement: Additional file 1: Table S1 — Organisms isolated in patients with suspected ventilator-associated respiratory infection; Table S2: Breakdown of diagnostic criteria for patients with sVARI. [file cc13077-S1.docx]

Supplemental Table 1: Organisms Isolated in Patients with Suspected Ventilator-associated Respiratory Infection

| Organism | Suspected VARI  (77) |
| --- | --- |
| Staphylococcus Aureus  MSSA  MRSA | 6  1  5 |
| Pseudomonas. aeruginosa | 4 |
| Klebsiella Sp. | 8 |
| Escherichia coli | 2 |
| Stenotrophomonas | 1 |
| Enterobacter Sp. | 4 |
| Pantoea Sp. | 3 |
| Aspergillus fumigatus | 1 |
| Candida albicans | 3 |
| Polymicrobial | 21 |
| Normal flora | 9 |
| Sample not obtained | 15 |

MSSA, methicillin-sensitive *Staphylococcus aureus*; MRSA, methicillin-resistant *Staphylococcus aureus.*

Supplemental Table 2: Breakdown of Diagnostic Criteria for Patients with sVARI

| Diagnostic Criteria combinations  n (%) | Suspected VARI  (77) |
| --- | --- |
| WBC + Temperature + CXR | 4 (5.2%) |
| WBC + Secretions + CXR | 19 (24.7%) |
| Temperature + Secretions + CXR | 3 (3.9%) |
| Temperature + Secretions + WBC + CXR | 3 (3.9%) |
| Temperature + Secretions + WBC | 48 (62.3) |

WBC, white blood cell count; CXR, chest X-ray
