# Supplementary material for: Suspected ventilator-associated respiratory infection in severely ill patients: a prospective observational study
Source: Crit Care. 2013 Oct 22;17(5):R251. doi: 10.1186/cc13077 (PMC4056611; doi:10.1186/cc13077)
Supplement: Additional file 2 — Frequency distribution of sVARI by days of mechanical ventilation. Data shown are a frequency distribution representing the number of incident sVARIs by duration of mechanical ventilation. [file cc13077-S2.pdf]

Supplementary Figure 1:

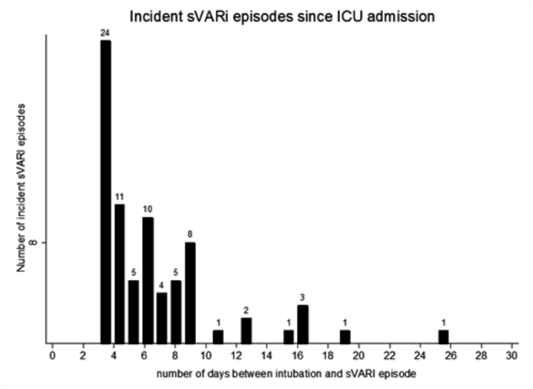

Supplementary Figure 1: A frequency distribution representing the number of incident sVARIs by duration of mechanical ventilation is shown.
